# Combining Linguistic, Behavioral and Visuospatial Measures to Characterize Multidomain Impairment in Dementia

**DOI:** 10.3390/brainsci16050511

**Published:** 2026-05-11

**Authors:** Renate Delucchi Danhier, Barbara Mertins

**Affiliations:** Psycholinguistics Laboratories, TU Dortmund University, 44227 Dortmund, Germany

**Keywords:** dementia, Alzheimer’s disease, cognitive decline, eye-tracking, visuospatial processing, language impairment, narrative production, multimodal assessment

## Abstract

**Highlights:**

**What are the main findings?**

- Dementia patients show consistent multidomain impairment, with the strongest differences observed in linguistic quality (reduced complexity, coherence, and increased errors) and visuospatial task performance (slower and less accurate execution).
- A multimodal profile combining linguistic, behavioral, and eye-tracking measures differentiates clinical and control groups at the sample level, while eye-tracking alone shows a weaker and less consistent pattern.

**What are the implications of the main findings?**

- Multimodal assessment captures cross-domain patterns of cognitive impairment that are not evident in single-domain measures, highlighting the value of integrating language and behavior in dementia characterization.
- Findings support the use of multimodal profiling as a research framework for studying heterogeneous cognitive decline and as a basis for future investigation in earlier disease stages.

**Abstract:**

**Background/Objectives**: Visuospatial impairments are among the earliest cognitive symptoms in Alzheimer’s disease (AD) and related dementias (ADRD), yet standard assessments often lack ecological validity and focus on isolated domains. This study examines whether integrating linguistic, behavioral, and eye-tracking measures provides a more comprehensive characterization of cognitive deficits within a multimodal, exploratory framework. **Methods**: Twenty older adults (10 with mild to moderate dementia, including AD/ADRD, and 10 age-matched controls) completed three tasks: (1) oral narrative production, (2) visuospatial behavioral tasks (manipulation, recognition, reproduction), and (3) free-viewing eye-tracking. Linguistic, behavioral (time, errors), and fixation-based measures were analyzed using non-parametric statistics, with emphasis on effect sizes and cross-domain patterns. **Results**: The clinical group differed consistently from controls across domains. Linguistic measures showed increased output but reduced quality, including lower syntactic complexity, more grammatical errors, greater pragmatic deviations, and reduced gist comprehension. Behavioral tasks revealed slower performance and more frequent failures. Eye-tracking differences were less pronounced, showing a tendency toward longer fixations and less efficient visual exploration. A composite multimodal index showed clear separation between groups, indicating a consistent pattern of impairment across measures. **Conclusions**: Cognitive differences in dementia are expressed across multiple domains, with the strongest effects in linguistic and behavioral measures. These findings highlight the value of multimodal profiles for capturing multidimensional impairment. Results should be interpreted as exploratory and require confirmation in larger, confirmatory studies.

## 1. Introduction

Dementia currently affects approximately 57 million people worldwide and is projected to reach 153 million by 2050, largely due to population aging [1]. Alzheimer’s disease (AD), the most prevalent form of dementia, accounts for approximately two-thirds of dementia cases and is characterized by progressive decline across multiple cognitive domains, including memory, language, executive functioning, and visuospatial abilities. Neuropathologically, AD is defined by the accumulation of β-amyloid plaques and tau neurofibrillary tangles, leading to widespread cortical degeneration. Given the absence of curative treatment, characterizing disease-related changes across cognitive domains remains a central challenge in dementia research, particularly Alzheimer’s disease.

Clinically, AD may present as early-onset (before age 65) or, more commonly, late-onset disease. Standard diagnostic procedures rely on neuropsychological screening tools such as the Mini-Mental State Examination (MMSE) and the Montreal Cognitive Assessment (MoCA). However, pathological processes begin years before clinically detectable symptoms emerge. During this prodromal phase, subtle cognitive and behavioral changes—often reported as Subjective Cognitive Impairment (SCI)—may remain undetected by standard instruments. Longitudinal evidence suggests that early markers, including linguistic features, can precede clinical diagnosis by decades [2]. These findings highlight the need for more sensitive and ecologically valid approaches for characterizing cognitive changes across disease stages. While these findings motivate research into early detection, the present study does not address preclinical stages and instead focuses on individuals with clinically established dementia.

### 1.1. Cognitive Markers of Dementia (With Emphasis on Alzheimer’s Disease)

#### 1.1.1. Linguistic Markers

Language impairments are among the earliest observable changes in AD. Patients often exhibit lexical retrieval difficulties, reduced vocabulary richness, increased use of pronouns and generic terms, and simplified grammatical structures [3,4,5,6]. Written language shows similar patterns, including shorter texts and increased errors [4,7].

While basic grammatical structures may appear preserved in early stages, impairments emerge in lexical-semantic processing, syntactic complexity, and discourse organization. For instance, AD patients tend to produce fewer subordinate clauses and shorter syntactic units, reflecting reduced linguistic complexity [8,9]. Discourse-level impairments include reduced global coherence and diminished ability to maintain thematic structure [10]. These findings suggest that language alterations in AD reflect both compensatory strategies and underlying cognitive decline.

#### 1.1.2. Behavioral and Visuospatial Markers

In addition to language, AD affects visuospatial processing due to degeneration in temporoparietal and occipital regions. These impairments manifest in difficulties with spatial orientation, object manipulation, and action planning [11,12].

Behavioral “act-out” tasks have been widely used to assess visuospatial and praxis abilities. Studies show that AD patients perform more slowly and less accurately in tasks such as gesture imitation and visuoconstructive activities, often displaying disorganized strategies and increased error rates [13,14,15,16]. These deficits are commonly interpreted as markers of impaired executive control and spatial processing.

#### 1.1.3. Eye-Tracking and Visual Attention

Eye-tracking provides a sensitive method for assessing visual attention and oculomotor control. AD has been associated with altered gaze behavior, including longer fixation durations, increased fixation counts, slower saccades, and less efficient visual search strategies [17,18,19,20].

However, findings are not entirely consistent. While some studies report pronounced impairments in attentional allocation and novelty detection [21], others suggest that basic oculomotor functions may remain relatively preserved, with deficits emerging primarily in higher-order, goal-directed viewing behavior. This divergence reflects ongoing debate regarding whether eye-tracking measures capture primary visuospatial deficits or broader impairments in attention and executive control.

### 1.2. Limitations of Single-Domain Approaches

Although standard neuropsychological batteries commonly used in dementia research assess multiple cognitive domains, they typically do so through separate, highly structured, and decontextualized tasks. This approach is clinically valuable for isolating specific functions, but it may not fully capture how language, visuospatial processing, attention, and goal-directed behavior interact during more naturalistic activities. In everyday contexts, these cognitive systems operate in an integrated manner, requiring individuals to coordinate multiple processes simultaneously. In dementia, impairments are often heterogeneous: patients may perform relatively well in some domains while showing marked difficulties in others. Consequently, assessments that treat domains independently may overlook cross-domain patterns that become apparent when linguistic, behavioral, and visual-attentional processes are examined together within the same task context.

Existing research has increasingly recognized the value of multimodal assessment [17,22], but many studies still examine language, behavior, or eye movements in isolation, or combine modalities at the level of classification rather than analyzing how they converge within the same participants across related tasks. This limits insight into how different cognitive systems interact within individuals. The present study addresses this gap by integrating narrative production, visuospatial task performance, and eye-tracking measures to characterize dementia-related impairment across complementary and ecologically relevant levels of behavior.

### 1.3. Multimodal Approaches to Dementia Assessment

A growing number of studies have explored multimodal approaches in dementia research, combining behavioral, linguistic, physiological, oculomotor and neuroimaging data. However, their contributions differ substantially with respect to reference standards, analytical integration, and ecological validity. In particular, it remains important to clarify whether multimodal combinations provide measurable improvements over established clinical benchmarks (e.g., clinical diagnosis or neuropsychological testing), and how such gains translate to real-world cognitive functioning.

Previous work combining behavioral and physiological modalities has demonstrated that multimodal data can improve discrimination between individuals with dementia and controls when compared to single-domain features. For example, studies integrating speech, motor behavior, and physiological measures have classified cognitive status relative to clinical diagnostic labels, showing improved classification performance when modalities are combined [22]. However, these studies typically use clinical diagnosis as the reference standard, and the extent to which multimodal models outperform standard cognitive assessments (e.g., MMSE or clinical evaluation) is not always clearly quantified. In addition, although multiple modalities are recorded, they are often analyzed in parallel rather than examined in terms of how different cognitive systems interact within the same individuals during task performance. Tasks also tend to remain relatively structured, limiting ecological validity.

Other approaches have combined neuroimaging with behavioral or virtual reality paradigms. For instance, integrating MRI-derived structural markers with eye-tracking measures in virtual navigation or visual search tasks has been shown to improve classification accuracy relative to imaging alone when predicting Alzheimer’s disease diagnosis [23]. While these findings suggest that multimodal combinations can enhance performance over single measures, the reference standard remains clinical diagnosis, and the incremental benefit over established neuropsychological testing is often not specified. Moreover, these paradigms are typically domain-specific (e.g., spatial navigation) and rely on specialized equipment, limiting their ability to capture interactions between language, behavior, and attention in more naturalistic contexts.

Recent studies have also combined facial expression analysis, linguistic performance, and eye-tracking measures using machine learning approaches to classify cognitive status relative to clinical categories [24]. In these cases, multimodal models often outperform single-modality approaches in terms of classification accuracy. However, performance is primarily evaluated relative to diagnostic labels, without systematic comparison to standard screening instruments or clinical judgment. Furthermore, modalities are frequently integrated at the level of statistical prediction rather than analyzed as interacting components of behavior within the same task context.

Taken together, existing multimodal approaches suggest that combining data sources can improve classification performance relative to single modalities. However, many studies lack clear benchmarks against standard clinical assessment, provide limited information on incremental value beyond established cognitive tests, and often do not examine how different cognitive domains interact within individuals during ecologically meaningful tasks. The present study addresses this gap by focusing on the convergence of linguistic, behavioral, and visual-attentional measures within the same participants and task contexts, with the aim of characterizing multidomain impairment rather than optimizing diagnostic classification. Although many prior studies are framed in terms of early or preclinical detection, the present work does not evaluate such applications and instead examines whether robust multimodal differences can be observed in clinically manifest dementia.

### 1.4. Aim of the Present Study

The present study adopts a multimodal approach combining linguistic analysis, visuospatial behavioral tasks, and eye-tracking measures to investigate cognitive markers associated with dementia, including AD and related dementias (ADRD). By comparing individuals with dementia to similarly aged healthy controls across multiple tasks, the study aims to identify consistent patterns of impairment across domains and to evaluate whether multimodal profiling enhances the characterization of cognitive impairment across domains in individuals with clinically manifest dementia.

We hypothesized that the clinical group would show reduced linguistic complexity and coherence, slower and less accurate behavioral performance, and less efficient visual exploration. Given the exploratory design, we further expected that no single measure would fully account for group differences, but that converging patterns across modalities would provide a more comprehensive characterization of impairment. The present study focuses on individuals with clinically established (mild-to-moderate) dementia and is not designed to evaluate preclinical or prodromal detection. Instead, it aims to establish whether multimodal profiling can capture coherent patterns of impairment across domains within symptomatic populations. Accordingly, the present study should be understood as an exploratory proof-of-concept investigation in clinically established dementia, providing a first step toward evaluating whether similar multimodal profiles may be informative in earlier or at-risk populations.

## 2. Materials and Methods

The present study employs a multimethod experimental design combining linguistic analysis, visuospatial behavioral tasks, and eye-tracking measures to investigate cognitive and perceptual indicators of dementia, including Alzheimer’s disease and related dementias (ADRD). The overarching goal is to compare individuals with clinically diagnosed dementia and age-matched healthy control participants with regard to language production, spatial reasoning, and visual exploration behavior. The focus is on measuring the same participants across diverse tasks, rather than trying to obtain a large sample. Three complementary tasks were administered:

1. Oral language production task (picture book);
2. Visuospatial behavioral tasks involving object manipulation;
3. Free viewing eye-tracking task.

The combination of linguistic and visuospatial paradigms allowed us to investigate both overt behavioral performance and implicit visual attention processes. This multimethod approach was designed to capture cognitive alterations associated with AD across different domains of language and spatial perception.

### 2.1. Participants

Participants (N = 20) consisted of ten individuals diagnosed with mild to moderate dementia (including Alzheimer’s disease and unspecified dementia diagnoses) and ten age-matched healthy elderly controls. Patients were recruited through dementia care homes and local Alzheimer’s associations. Recruitment relied on voluntary participation from residents. Diagnoses were based on prior clinical evaluation by medical professionals. Four clinical participants had a diagnosis of comorbid depression at the time of testing. Control participants reported no neurological or psychiatric disorders. All participants were native monolingual speakers of German living in Germany. Especially in the eye-tracking task, data quality was variable because of calibration problems and a low tracking ratio. Table 1 gives an overview of age, gender and diagnosis of the participants in each group.

Dementia diagnoses in the clinical group were established prior to the study by qualified medical professionals in the care institutions as part of routine clinical assessment. Although detailed diagnostic protocols were not available for the present study, diagnoses were based on standard clinical procedures commonly used in geriatric practice in Germany, including neuropsychological assessment tools such as the Mini-Mental State Examination (MMSE) and the Montreal Cognitive Assessment (MoCA). No biomarker-based confirmation (e.g., CSF analysis, PET imaging, or MRI-based diagnostic classification) was available for the present sample. Diagnostic labels included Alzheimer’s disease, unspecified dementia, and senile dementia, reflecting the heterogeneity of clinical diagnoses in real-world care settings. Due to the limited availability of detailed etiological information and the small sample size, participants were analyzed as a mixed dementia group rather than as separate diagnostic subgroups.

Control participants were recruited from the same residential settings and had no documented diagnosis of dementia or other neurological disorders. The absence of dementia was based on routine yearly clinical evaluations conducted within the care institutions, which included non-pathological neuropsychological assessment via MMSE/MoCA within less than one year prior to testing. No additional independent cognitive screening was performed as part of the present study.

Group equivalence on demographic variables was assessed descriptively and using non-parametric tests. Age distributions were comparable between groups, with no indication of a systematic difference (Mann–Whitney U = 43.0, *p* = 0.62, r ≈ 0.11). Gender distribution differed descriptively between groups (clinical: 8 female, 2 male; control: 4 female, 6 male), although this difference was not statistically significant (Fisher’s exact test, *p* ≈ 0.17). Given the small sample size, these demographic comparisons are interpreted cautiously.

The sample sizes are indeed relatively small because of practical challenges associated with recruiting individuals diagnosed with Alzheimer’s disease or older participants in general. Such small sample sizes are common in studies involving older clinical populations and mobile eye-tracking setups. No formal a priori power calculation was conducted because the study was designed as an exploratory, hypothesis-generating investigation rather than a confirmatory trial. The sample size was constrained by the practical challenges of recruiting older participants with clinically diagnosed dementia and by the intensive multimodal protocol, which required each participant to complete narrative, visuospatial behavioral, and eye-tracking assessments. This design prioritizes detailed within-participant characterization across multiple domains over large-scale statistical inference. Accordingly, the analyses focus on effect sizes, direction and consistency of effects, and convergence across modalities rather than on definitive hypothesis testing. The findings should therefore be interpreted as preliminary and require replication in larger, adequately powered samples. Therefore, the present study adopts an explicitly exploratory approach, focusing on identifying converging patterns across modalities rather than testing a small number of predefined hypotheses. Our aim is to identify how linguistic, behavioral and visual markers associated with dementia, including Alzheimer’s disease and related dementias, manifest in the same patients and to assess whether similar patterns occur in healthy individuals. These insights may guide future studies with larger samples and confirmatory statistical designs. The resulting sample sizes are comparable to those reported in previous eye-tracking studies investigating visual attention and language processing in Alzheimer’s disease populations [25,26,27] and related dementias.

### 2.2. Materials and Procedure

#### 2.2.1. Linguistic Task

To assess linguistic abilities, participants were tasked with orally narrating a modified version of the wordless picture book “Frog, Where Are You?”(Dial Books for Young Readers, New York, NY, USA) [28], a widely used stimulus in psycholinguistic research. To reduce cognitive load, the original 24-page story was shortened to 11 pages while preserving the storyline. Images were enlarged and colored to improve visual contrast and accommodate age-related visual impairments. To avoid influencing narration, experimenters intervened only after extended pauses, using neutral prompts (e.g., “What else is happening?”). Because the pictures are physically present during the narration, this minimizes memory overload (specifically episodic memory), allowing a better isolation of syntactic or semantic language decline.

An utterance was defined as a sequence of words forming a syntactic unit, typically containing no more than one finite verb, and served as the primary unit of analysis. Because participants differed substantially in the total number of utterances produced, linguistic variables were normalized relative to total utterances to ensure comparability across individuals. Task adherence was assessed by the proportion of pragmatically unrelated utterances, defined as instances in which participants deviated from the narrative and talked about personal experiences instead of retelling the story.

#### 2.2.2. Visuospatial Behavioral Tasks

To assess visuospatial abilities, participants completed three non-verbal behavioral tasks with no time limit, although participants were told to complete them as quickly as possible. Between tasks, there was a short resting pause. The three behavioral tasks were always performed in this order. The fixed order was chosen to ensure a structured progression of task demands and to avoid abrupt changes in difficulty.

**Figure 1:**
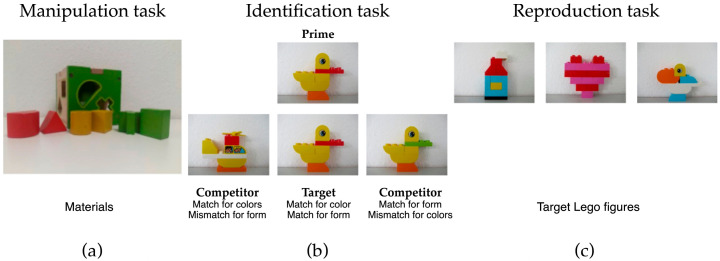
Materials and stimuli for the visuospatial behavioral tasks. (**a**) Object manipulation task, (**b**) Object recognition task, (**c**) Object reproduction task.

1. Object manipulation task: Using a standard children’s shape-sorting toy, participants inserted six wooden geometric shapes into a wooden box through the corresponding openings (Figure 1, left);
2. Object recognition task (3 items): Participants viewed a photograph of an object constructed from Lego Duplo bricks and then selected the identical object from three visually similar alternatives. (Figure 1, middle);
3. Object reproduction task (3 items): This task placed higher demands on visuospatial planning and execution. Participants had to build a copy of an already built Lego Duplo figure with the loose bricks provided to them (Figure 1, right). A trial was considered forfeited if the participant themselves considered they could not come to a solution they considered satisfactory. Participants had to clearly express verbally that they had given up. When a participant’s solution did not match the target object, this was counted as an error. Thus, participants were aware of forfeits, but not necessarily of errors.

The object recognition task was grouped under visuospatial behavioral measures, as successful performance requires the processing of visual form and spatial features. However, it is important to note that this task is not purely visuospatial. Accurate recognition also depends on intact semantic knowledge and object representations, as well as broader cognitive processes such as attention and decision-making. Accordingly, performance on this task may reflect a combination of visuospatial processing, semantic memory, and higher-level cognitive functions rather than a single domain in isolation.

#### 2.2.3. Eye-Tracking Tasks

**Figure 2:**
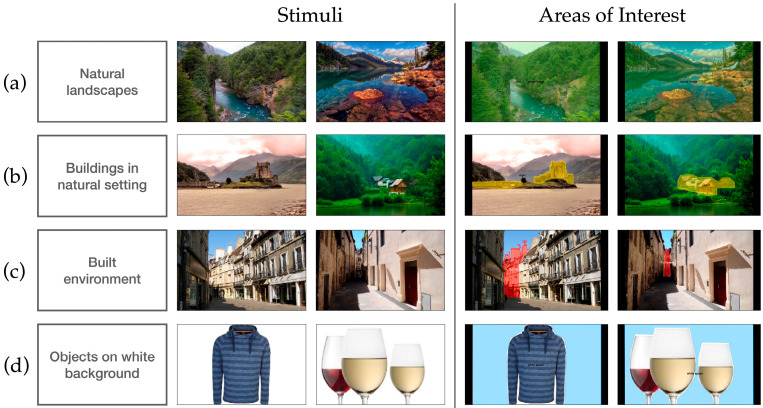
Photographs and areas of interest for the eye-tracking tasks. (**a**) Visual exploration behavior, (**b**) Attention to foreground, (**c**) Vanishing point identification, (**d**) Disregard of white space.

To assess participants’ implicit visual processing of spatial information, we designed three eye-tracking tasks in which the evaluation criteria were not disclosed. This allowed us to examine whether spontaneous viewing behavior aligned with typical patterns observed in healthy individuals. Six clinical photographs were presented (Figure 2). Each clinical trial was preceded by a central fixation cross (trigger AOI) to ensure a standardized starting position. Six distractor images (e.g., food or drinks) were interleaved with the clinical items. In total, the eye-tracking task had a duration of around three minutes (taking into consideration calibration and the variable time to trigger the next image), making it a quick and non-invasive assessment method.

1. Visual exploration behavior: In two pictures of natural landscapes, we measured abnormally long fixations as an index of reduced visual exploration. A fixation was classified as abnormally long if its duration exceeded the median of the control group fixation durations by more than 1.5 times the interquartile range (IQR). Based on the present data for the control group, taking all fixations in the two natural landscapes (median = 249.9 ms, IQR = 166.6 ms), this corresponded to a threshold of approximately 500 ms. Fixation durations in natural viewing and reading tasks typically center around 200–300 ms and exhibit a right-skewed distribution with a long tail of extended fixations [29,30]. While most fixations fall within this typical range, durations can extend beyond 500 ms, representing the upper tail of the distribution and commonly reflecting increased cognitive processing demands or attentional difficulty [30,31]. This non-parametric criterion provides a robust and data-driven definition of unusually long fixation events.
2. Attention to foreground: In two scenes depicting built elements in natural settings, we assessed if the buildings were fixated. To determine whether AD patients are less driven by novelty or informativeness and distribute attention more evenly rather than prioritizing relevant scene components [21] we defined areas of interest (AOIs) over the built elements in the castle and cabin images. To operationalize participants paying attention to the foreground, the entry times into these AOIs were measured.
3. Vanishing point identification: Because urban photographs contain dense visual information and rely strongly on geometric structure and depth cues, they were expected to place particular demands on visuospatial processing. For the two images of urban environments, areas of interest were defined over the region with the greatest visual depth. To operationalize participants identifying the vanishing point, we measured if the participants fixated on the vanishing point area of interest. Figure 2c shows the region with the greatest visual depth. Urban scenes depend heavily on visuospatial organization, geometry, alignment, and depth. Since dementia affects temporoparietal and occipital systems involved in visuospatial processing, these areas of interest should be especially informative.
4. Disregard of white space: To check if the participants manage to keep their visual attention on pictorial elements, big objects were presented in the center of the screen. We measured whether participants fixated on the uninformative white space, the area of the photograph not covered by the object.

During filler trials, participants orally answered simple questions to maintain engagement. Each image was presented for 10 s. Eye movements were recorded with a remote SMI eye-tracker (60 Hz) while stimuli were presented on a 24-inch (16:9) monitor. Participants were seated ~65–70 cm from the monitor, with individually adjusted monitor height. A five-point calibration was performed and repeated, if necessary, to ensure accuracy. Participants were instructed to look at the images and respond orally to auditory questions. All experimental sessions took place in familiar environments within the participants’ care institutions in order to minimize stress and ensure participant comfort. Sessions typically began with an informal conversation to establish rapport before the experimental tasks were introduced.

Eye-tracking data were analyzed using fixation-based measures to assess patterns of visual exploration and attentional allocation. Fixations were defined as gaze maintained within a spatial dispersion of 1.0° for ≥100 ms. Analyses were based on the eye with better calibration accuracy. Eye-tracking data quality was evaluated using tracking ratio and calibration accuracy measures. In line with established practice in eye-tracking research, tracking ratios above approximately 70% are generally considered acceptable for fixation-based analyses, while calibration errors below 1–1.5° are regarded as optimal, and errors exceeding 3° indicate reduced spatial accuracy that may affect area-of-interest–based measures. In the present dataset, a majority of participants met acceptable quality standards; however, some recordings showed reduced tracking ratios (e.g., D06 and H09 < 60%) or elevated calibration errors (e.g., D05 > 3°), indicating increased variability in data quality, particularly within the clinical group. Given the exploratory nature of the study and the limited sample size (N = 10 per group), these thresholds were not used as strict exclusion criteria in order to preserve statistical power and avoid selective bias. Instead, all participants were retained in the primary analyses. To assess the robustness of the findings with respect to data quality, sensitivity analyses were conducted excluding participants with low-quality recordings (defined as tracking ratio < 60% or calibration error > 3° in either dimension). These analyses allow evaluation of whether the observed patterns are driven by a small number of low-quality datasets.

### 2.3. Hypotheses

Given previous evidence on linguistic, visuospatial, and oculomotor impairments in Alzheimer’s disease and related dementias, we expected the clinical group to differ from age-matched healthy controls across all three domains examined in the present study. More specifically, we hypothesized that participants in the clinical group would show (1) reduced linguistic performance, reflected in lower syntactic complexity, as well as more grammatical and pragmatic deviations; (2) reduced visuospatial behavioral performance, reflected in slower and less accurate execution of behavioral tasks; and (3) altered visual exploration patterns during the eye-tracking task, reflected in less efficient allocation of visual attention to task-relevant spatial information.

Since the study was designed as an exploratory multimodal investigation, we did not assume that all markers would differentiate the groups equally strongly. Rather, we expected the combined analysis across linguistic, behavioral, and eye-tracking measures to provide a more comprehensive characterization of dementia-related impairment than any single measure in isolation. A composite index was constructed post hoc to summarize cross-domain patterns and was not pre-specified.

## 3. Results

Given the exploratory nature of the study and the relatively small sample size, statistical analyses were conducted with the primary aim of identifying patterns of group differences across modalities rather than testing a limited set of confirmatory hypotheses. Group comparisons were performed using non-parametric Mann–Whitney U tests for continuous variables and Fisher’s exact tests for categorical variables.

Effect sizes (r) are reported alongside test statistics to provide an estimate of the magnitude of group differences. Interpretation of results is based primarily on the consistency and direction of effects across measures and domains, rather than on individual *p*-values.

Because multiple outcome measures were analyzed across linguistic, behavioral, and eye-tracking domains, the risk of inflated Type I error was considered. To address this, no single test was treated as confirmatory. Instead, the overall pattern of results across modalities was emphasized.

As a sensitivity analysis, *p*-values were additionally adjusted using the Benjamini–Hochberg procedure to control the false discovery rate (FDR) within each domain to evaluate the robustness of observed patterns. A nominal significance threshold of α = 0.05 is reported for descriptive purposes only and is not used as a strict decision criterion.

### 3.1. Linguistic Results 

Participants’ retellings of the “Frog Story” were analyzed with respect to both structural and content-related linguistic measures. Linguistic measures were analyzed at the utterance level (see Section 2.2.1).

Syntactic complexity was assessed by the proportion of subordinate utterances. Grammatical accuracy was evaluated by the proportion of utterances containing syntactic or morphological errors, serving as an indicator of linguistic impairment. Task adherence was assessed via pragmatically unrelated utterances (see Section 2.2.1). Finally, a semantic–pragmatic measure captured whether participants identified the central theme (“gist”) of the story, namely that the narrative revolves around a boy searching for a frog.

Because linguistic data were coded manually, inter-rater reliability was assessed on 50% of the dataset, which was independently annotated by two raters (5 clinical and 5 control participants). Following the calculation of Cohen’s κ (κ = 0.92), discrepancies were reviewed and resolved to establish a shared coding framework. The remaining 50% of the data was subsequently coded individually, with each rater annotating half of the dataset based on the agreed criteria. Table 2 gives an overview of all linguistic variables for all participants for the narrative task.

Given the skewed distributions and the presence of outliers (particularly among the clinical group), group comparisons were conducted using non-parametric tests. Across measures, a consistent pattern of differences emerged between groups:

Participants in the clinical group produced a higher number of utterances overall (U = 22.00, *p* = 0.019, r = 0.48), indicating greater verbal output. In contrast, the proportion of subordinate utterances was higher in the control group (U = 24.00, *p* = 0.027, r = 0.45), suggesting greater syntactic complexity.

The clinical group also showed higher proportions of grammatically incorrect utterances (U = 18.00, *p* = 0.010, r = 0.53) and pragmatically unrelated utterances (U = 13.50, *p* = 0.004, r = 0.62), reflecting reduced grammatical accuracy and task adherence. These effects were moderate to large in magnitude within this sample. At the discourse level, participants in the control group more frequently captured the central theme of the story (9/10) compared to the clinical group (3/10; Fisher’s exact test *p* = 0.019), indicating differences in global coherence and thematic integration. One control participant also failed to identify the gist of the story, indicating that this measure is not fully specific to the clinical group and should be interpreted as a coarse discourse-level indicator rather than a diagnostic marker.

Taken together, these results suggest a dissociation between quantity and quality of language in the clinical group: while verbal output was higher, it was accompanied by reduced structural complexity, increased error rates, and greater pragmatic deviation (higher quantity but lower quality of language), alongside a reduced ability to capture the gist of the story. This pattern was consistent across multiple linguistic measures, supporting the interpretation of a robust cross-measure difference rather than reliance on any single statistical test. FDR-adjusted results within the linguistic domain showed a comparable pattern, with all effects remaining below the conventional threshold. This interpretation is further evaluated in Section 3.4 using a multimodal composite measure.

### 3.2. Behavioral Results

Behavioral performance was assessed across three visuospatial tasks: object manipulation, object recognition, and object reproduction. Given the exploratory design and the number of related outcome measures (e.g., time, errors, and composite indicators), results are interpreted with a focus on consistency of patterns within and across tasks.

#### 3.2.1. Object Manipulation

Table 3 shows the time (measured in seconds) participants needed to insert the six geometrical forms into the wooden toy through the correct holes, as well as the number of mistakes they made while doing so.

Errors were rare, with only one participant (clinical group) making mistakes, indicating that accuracy was near the ceiling in both groups. In contrast, completion time showed a clear group difference. Participants in the clinical group required more time to complete the task (*U* = 15.00, *p* = 0.011, *r* = 0.57), with substantially greater variability (range = 18–457 s) compared to the control group (range = 24–140 s). The effect size was large, suggesting reduced efficiency and increased variability in task execution in the clinical group. Notably, individual variability was substantial, with one participant in the clinical group showing faster performance than all controls.

#### 3.2.2. Object Recognition

Table 4 shows how long participants took to recognize the target Lego object out of the triad from alternatives and their mistakes.

Error rates were low in both groups and did not show a clear group difference. Completion time again showed a consistent pattern, with participants in the clinical group requiring more time than controls (*U* = 23.00, *p* = 0.021, *r* = 0.48). The effect size was moderate to large. In addition to slower performance, the clinical group exhibited greater variability (range = 6–168 s) than participants in the control group (range = 4–58 s), indicating less stable task execution.

#### 3.2.3. Object Reproduction

Forfeits, errors and the time each participant needed to complete the three reproduction tasks for both groups are summarized in Table 5.

Across multiple indicators (including completion time, errors, and forfeitures), a consistent pattern of reduced performance was observed in the clinical group: Participants in the clinical group required more time to complete the task (*U* = 21.00, *p* = 0.016, *r* = 0.50) and produced more errors (U = 20.50, *p* = 0.017, r = 0.51). Forfeitures occurred only in the clinical group (4/10 participants), although this difference should be interpreted cautiously given the small sample size. A composite measure of total failures (errors + forfeitures) also showed higher values in the clinical group (U = 16.50, *p* = 0.012, r = 0.55). As this measure is derived from its components, it is not statistically independent and is therefore interpreted as a summary performance indicator rather than as an additional independent test. Controls are qualitatively mostly error-free and forfeit free. The failures can be qualitatively categorized as shown in Table 6.

Some participants in the clinical group performed comparably to the participants in the control group, including three patients who completed the task without any failures and especially D02, which was quicker than the mean of the controls.

#### 3.2.4. Summary of Behavioral Findings

Across all three tasks, a consistent pattern emerged in which the clinical group showed slower performance and (in more demanding tasks) increased error rates and task failures. Time-based measures were particularly sensitive in differentiating groups, while error rates showed clearer differences in tasks with higher cognitive demands.

Importantly, these patterns were observed across multiple related measures within each task, supporting the interpretation of reduced efficiency and accuracy in visuospatial task performance in the clinical group. Given the number of comparisons and the exploratory design, emphasis is placed on the convergence of effects across measures rather than on individual statistical tests. Within the behavioral domain, FDR-adjusted results similarly preserved the overall pattern, particularly for time-based measures (See Section 3.4).

Although the reproduction task, administered last, showed the largest group differences, group effects were already present in earlier behavioral measures, suggesting that the observed pattern cannot be attributed solely to order-related fatigue. Instead, the increasing magnitude of differences is consistent with the higher cognitive demands of later tasks, which may amplify underlying impairments.

### 3.3. Eye-Tracking Results

Eye-tracking data quality was assessed using tracking ratio and calibration accuracy (Table 7). Some variability in data quality was observed, particularly in the clinical group, where calibration was less stable. Tracking ratios, however, were comparable across groups, suggesting that overall data usability was sufficient despite increased variability.

Calibration was more difficult with the clinical group than for controls, but the tracking ratio was actually higher for the clinical group, with comparable variability across groups. Dementia patients had a higher mean calibration compared to healthy controls in both x-dimension and particularly in the y-dimension, suggesting less consistent calibration performance, driven by some extreme values within the dementia sample.

Fixation-based measures were analyzed (see Section 2.2.3). Across eye-tracking measures, group differences were less consistent than in the linguistic and behavioral domains, with effects varying depending on the specific aspect of visual processing assessed.

#### 3.3.1. Visual Exploration Behavior

The number of abnormally long fixations (defined as fixation durations ≥ 500 ms; see Section 2.2.3) is shown in Table 8.

Participants in the clinical group showed a tendency toward a higher number of abnormally long fixations compared to controls (U = 35.5, *p* = 0.29, r = 0.24), although this difference was not statistically pronounced. The effect size was small, and variability within the clinical group was substantial (range = 0–11), indicating substantial individual variability.

**Figure 3:**
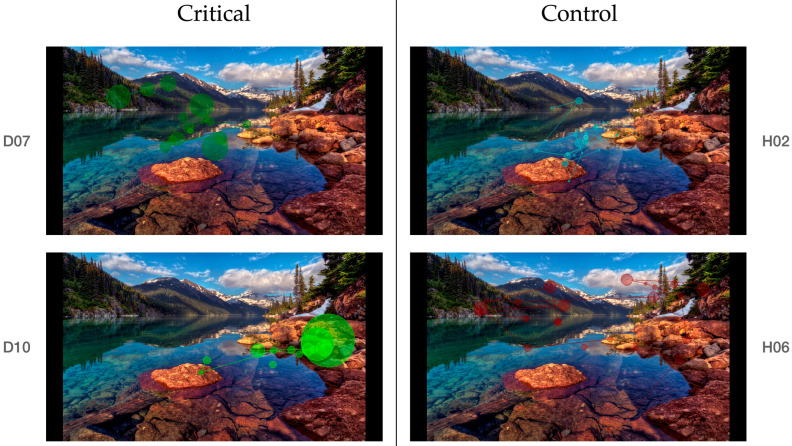
Scanpaths of the two participants per group with the highest number of long fixations for the ‘Canada’ stimulus. Fixations are represented by circles, where the area of the circle corresponds to fixation duration.

Longer fixation durations are commonly interpreted as reflecting slower processing or increased cognitive effort. In the present data, some clinical participants exhibited increased numbers of prolonged fixations, but this pattern was not consistently observed across the group and showed considerable overlap with control participants. Some of the longest fixation durations were observed in the clinical group, as can be seen in Figure 3.

#### 3.3.2. Attention to Foreground

Entry times into predefined areas of interest (AOIs) are presented in Table 9.

While control participants showed faster entry times on average, differences between groups were variable and did not show a consistent pattern (*U* = 32.00, *p* = 0.19, *r* = 0.29). The clinical group displayed markedly greater variability, including several extreme values, suggesting less consistent prioritization of relevant visual information. However, overlap between groups was substantial, and this measure did not clearly differentiate conditions.

#### 3.3.3. Vanishing Point Identification

Table 10 summarizes whether participants fixated on AOIs corresponding to the vanishing point in urban scenes.

Both groups showed generally high rates of fixation on these regions, with only small differences between groups (*U* = 32.50, *p* = 0.18, *r* = 0.30). Notably, only participants in the clinical group failed to fixate the vanishing point in both items, suggesting reduced consistency in attending to global spatial structure in a subset of individuals. However, overall group differences were modest, and this measure did not provide strong discrimination between conditions.

#### 3.3.4. Disregard of White Space

Fixations on white-space AOIs are summarized in Table 11.

Fixations on white space were comparable between groups (*U* = 47.50, *p* = 0.84, *r* = 0.05). Both groups showed similar patterns of gaze allocation, indicating that general attention to irrelevant regions did not differ meaningfully.

#### 3.3.5. Summary of Eye-Tracking Findings

In contrast to the more robust differences observed in linguistic and behavioral domains, eye-tracking measures showed weaker and less consistent group separation. While some tendencies toward longer fixation durations and less efficient allocation of visual attention were observed in the clinical group, these effects showed substantial overlap between groups and did not reliably differentiate conditions.

Sensitivity analyses excluding participants with low-quality eye-tracking recordings (D05, D06, and H09) did not materially alter the overall interpretation of the eye-tracking results. Across measures, the overall pattern remained unchanged. For long fixations, the effect became weaker (U = 29.5, *p* ≈ 0.44, r ≈ 0.19), while for entry times into foreground areas, the effect size increased slightly but remained non-significant (U = 24, *p* ≈ 0.14, r ≈ 0.36), with continued overlap between groups. Results for vanishing point identification were virtually unchanged (U = 27, *p* ≈ 0.18, r ≈ 0.32), and no group difference was observed for white space fixation (U = 36, *p* ≈ 1.00, r = 0.00). Effect sizes remained small to moderate and non-significant across measures, indicating that the limited discriminative value of the eye-tracking variables is not driven solely by data quality issues.

Overall, these findings suggest that eye-tracking measures provide complementary but comparatively less sensitive information in the present paradigm and should be interpreted in conjunction with linguistic and behavioral results rather than as standalone indicators.

### 3.4. Control for Multiple Testing (FDR)

To evaluate the robustness of these findings with respect to multiple comparisons, false discovery rate (FDR)–adjusted *p*-values were computed within each domain (Table 12).

All effects in the linguistic domain remained below the conventional threshold after FDR adjustment. This is also true for results in the behavioral domain, indicating that these patterns are comparatively robust within this dataset. In the eye-tracking domain, none of the measures fell below the conventional threshold after FDR correction, consistent with the generally weak and heterogeneous pattern observed in these variables.

### 3.5. Multimodal Integration: Converging Evidence Across Domains

**Figure 4:**
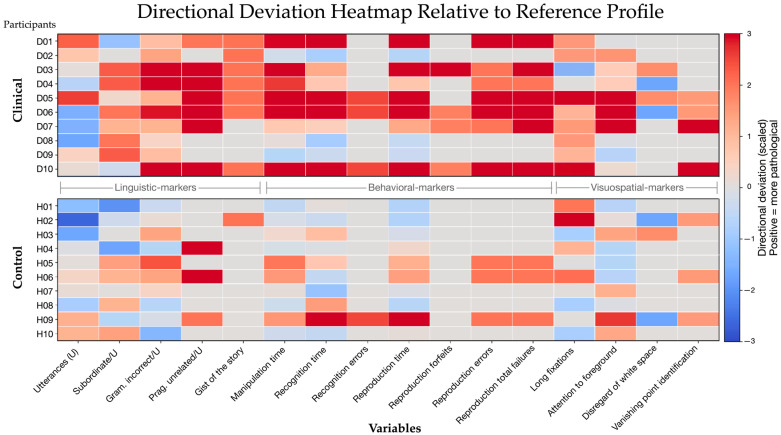
Multimodal heatmap of directional deviations and magnitude from the reference profile across linguistic, behavioral, and eye-tracking measures. Each cell represents the scaled deviation of an individual participant’s value from the reference value for that variable (the median of the control group). Color intensity reflects the magnitude and direction of deviation, with red indicating values in the pathological direction (based on predefined markers) and blue indicating values in the healthy direction. Values are normalized within each variable using robust scaling to allow comparability across measures. Participants are grouped by condition (clinical vs. control).

The central aim of the present study was to examine whether cognitive differences between groups can be characterized more clearly when multiple domains are considered jointly. Given the heterogeneity of dementia, individual participants may show relatively preserved performance in one task while exhibiting impairments in another. Figure 4 provides an overview of performance across linguistic, behavioral, and eye-tracking measures for each participant.

The visualization highlights a systematic tendency toward group-level divergence: participants in the clinical group show more frequent deviations from the reference profile, particularly in linguistic and behavioral measures, whereas control participants cluster more closely around baseline values. These differences are not driven by a single variable but emerge across multiple measures capturing distinct aspects of cognition.

Among the linguistic markers, the ones that discriminate the best between groups are the variables “pragmatic deviations” and “the gist of the story”. For the behavioral data, the most informative variables are “time to reproduce the Lego figures” and “failure to reproduce the Lego figures (forfeits plus errors)”, along with “time to recognize the target pictures”. Within the eye-tracking variables, unnaturally long fixations showed the clearest tendency toward group differences, although effects were weak and variable. The strongest group separation is observed in variables reflecting higher-order cognitive processing, including pragmatic coherence, error rates, and task performance measures. In contrast, eye-tracking variables display more heterogeneous and less pronounced deviations across groups, suggesting relative preservation of basic visual exploration.

Overall, the results indicate that impairments in the clinical group extend across multiple domains rather than being confined to isolated measures. The observed pattern suggests that cognitive changes in dementia affect several interacting systems, with particularly clear differences in tasks requiring structured processing and goal-directed behavior.

To summarize the extent to which deviations accumulate across domains within individuals, a composite “distance-to-reference” index was calculated by counting the number of variables on which each participant deviated from the reference profile in the predefined pathological direction. The reference was defined as the median of the control group (Table 13). This composite measure is derived from the same set of variables that were analyzed individually and is therefore not statistically independent of the preceding analyses. As a consequence, inferential statistical tests applied to this index do not provide additional confirmatory evidence beyond the individual tests, and the resulting effect size should be interpreted with caution. The composite index is used here as a descriptive summary of the accumulation of cross-domain deviations within individuals, rather than as an independent statistical test of group differences.

Because the composite index aggregates directional deviations rather than effect magnitudes, it should be interpreted as a coarse indicator of distributed impairment rather than a precise quantitative measure. A Mann–Whitney U test yields a moderate effect (U = 24.5, *p* ≈ 0.045, r ≈ 0.45); however, this result is expected given the aggregated construction of the index and should not be interpreted as independent statistical evidence.

From a descriptive perspective, however, the index illustrates a clear pattern: participants in the clinical group tend to show a higher number of deviations across measures compared to controls, reflecting the broad distribution of impairments across measures. This composite measure provides a convenient descriptive summary of group differences across measures, rather than a statistically independent indicator. Given the number of comparisons conducted across domains, single-measure results are inherently variable and should be interpreted cautiously. In contrast, the multimodal index captures the accumulation of small and moderate effects across measures, reducing the influence of isolated fluctuations and providing an integrated representation of overall cognitive differences.

Although some overlap between groups is present (e.g., one control participant showing higher deviation), the overall separation is visible, supporting the notion that dementia-related differences manifest as patterns spanning multiple measures rather than isolated deficits. It is important to note that the clinical group of dementia patients often showed a lot of inter-group variability. This pattern highlights the value of multimodal approaches, as emphasized in previous work [22,23], which demonstrates that combining different types of data can improve the detection and characterization of cognitive decline.

The results show that impairments in the clinical group are most pronounced in tasks requiring structured processing, integration of information, and goal-directed behavior. In contrast, more basic perceptual and oculomotor processes appear relatively intact. This dissociation suggests that dementia-related processes, particularly in Alzheimer’s disease, affect higher-order cognitive control mechanisms before more fundamental sensory processes. The results indicate that group differences are most clearly reflected in distributed patterns across measures. Rather than relying on single indicators, the present findings support an interpretation at the level of cross-domain profiles, with linguistic and behavioral measures contributing most strongly and eye-tracking measures providing complementary but less consistent information.

The primary evidence for multimodal convergence is therefore visual and pattern-based (Figure 4), rather than inferential at the level of the composite index. This pattern is consistent with models of distributed neurocognitive decline in dementia, in which impairments emerge across interacting systems rather than within isolated domains. In conclusion, the multimodal pattern emerges despite the variability and limited robustness of individual measures, reinforcing the value of integrated analyses in small-sample exploratory designs.

## 4. Discussion

We employed a multimodal approach to investigate dementia-related markers by integrating linguistic, behavioral, and eye-tracking measures. The main result is the multimodal profile that shows clear differences between groups; only one control participant (H09) overlaps with the clinical range. This type of integrated measure is what other studies have argued for [22,23].

The results reveal a consistent pattern of impairment in the clinical group across linguistic and behavioral domains, whereas eye-tracking measures showed more subtle differences. Taken together, these findings support the view that dementia affects multiple cognitive systems simultaneously and that a multimodal framework is well suited to capture this complexity.

Recent work highlights the potential of such approaches. For example, one study used a VR-based eye-tracking paradigm [23] combining visuospatial processing, attention, and memory within a single gaze-driven task while requiring minimal motor interaction, making it particularly suitable for older populations. More broadly, other studies integrating multiple modalities [22] demonstrate that combining behavioral, linguistic, and physiological measures can improve the detection of dementia-related patterns. In line with these findings, the present results suggest that multimodal assessment provides a more comprehensive characterization of cognitive impairment than single-domain approaches.

### 4.1. Linguistic Impairments: Reduced Complexity and Coherence

The linguistic findings align closely with previous research on language decline in dementia, particularly in Alzheimer’s disease. Although participants in the clinical group produced a higher number of utterances, this increased verbal output was accompanied by reduced syntactic complexity, a higher proportion of grammatical errors, and greater pragmatic deviation from the communicative task. This dissociation between quantity and quality of speech is consistent with evidence that individuals with dementia often produce more “empty speech” and rely on compensatory strategies, such as simpler structures and less informative language [8,9,32].

The reduced proportion of subordinate utterances supports the notion that individuals with dementia, particularly Alzheimer’s disease, often avoid complex syntactic constructions, which is likely to reduce cognitive load on working memory. This finding is consistent with previous work reporting shorter and less complex utterances and reduced dependency distance in syntactic structures [8,9]. The increased rate of grammatically incorrect utterances further indicates a decline in linguistic accuracy, which may reflect impairments in lexical retrieval and morphosyntactic processing.

At the discourse level, the higher proportion of pragmatically unrelated utterances as well as the reduced ability to capture the gist of the story indicate impairments in global coherence and thematic integration. This pattern is consistent with previous findings indicating that discourse produced by individuals with AD is less coherent and less closely aligned with the visual stimulus [10]. The picture-based narrative task reduces the need for episodic memory retrieval, as the visual stimuli remain available throughout the task. This design was adopted to help limit memory-related confounds (compared to tasks requiring recall of previously presented material). However, the task still places demands on working memory, attentional control, and executive processes, as participants must integrate information across successive images and maintain a coherent narrative structure. In addition, successful task performance requires sustained goal-directed behavior, including the ability to track the storyline across images. Therefore, while the task reduces episodic memory load, it does not isolate language processing from broader cognitive functions. The observed deficits are thus unlikely to be explained solely by episodic memory impairment and instead point to broader disruptions in language processing and discourse organization. These interpretations are supported by the consistency of effects across multiple linguistic measures rather than reliance on any single statistical comparison.

### 4.2. Behavioral Impairments: Reduced Efficiency and Accuracy

The behavioral findings further support the presence of visuospatial and executive impairments in the clinical group. Across all behavioral tasks, participants in the clinical group performed more slowly and produced more errors than controls. These results are consistent with previous studies demonstrating impaired performance in act-out and visuoconstructive tasks in Alzheimer’s disease and related dementias [16,33]. The longer completion times observed in the clinical group suggest reduced processing efficiency and increased cognitive effort. At the same time, higher error rates and the occurrence of task forfeitures indicate difficulties in planning, executing, and monitoring goal-directed actions. Such patterns are characteristic of apraxia and executive dysfunction, which are commonly reported in dementia, particularly Alzheimer’s disease [13,15]. Interpretation of performance in the object recognition task should be made with caution, as successful performance requires the coordination of visuospatial processing, semantic knowledge, and executive control. Consequently, deficits in this task cannot be attributed to a single domain in isolation, but may reflect impairments across multiple interacting cognitive systems.

In addition to these quantitative differences, qualitative differences between groups were observed. Participants in the clinical group more frequently showed disorganized construction strategies and inappropriate use of materials, indicating impairments not only in execution but also in problem-solving and spatial organization. These findings suggest that deficits extend beyond slowed performance and reflect disruptions in higher-order cognitive processes underlying visuospatial behavior, broadly consistent with patterns of posterior cortical involvement reported in dementia. These differences were observed across multiple related measures within each task, supporting the interpretation of reduced efficiency and accuracy at a broader behavioral level.

### 4.3. Eye-Tracking: Subtle Differences in Visual Exploration

The eye-tracking paradigm employed in this study was based on free-viewing of naturalistic scenes, comparable to exploratory designs used in previous work [21,27]. Such paradigms aim to capture spontaneous visual exploration without imposing explicit task demands, thereby providing insight into natural attentional allocation.

Based on prior research, we expected participants in the clinical group to exhibit less efficient visual exploration, reflected in longer fixation durations, an increased number of prolonged fixations, and reduced prioritization of informative regions such as foreground objects or spatially important elements such as the vanishing point. These expectations are grounded in evidence linking Alzheimer’s disease to impairments in visuospatial processing, attentional control, and visual search efficiency [17,18,19,20,21].

However, these predictions were only partially supported. Compared to the clear group differences observed in linguistic and behavioral domains, eye-tracking measures showed weaker and less reliable differentiation. Although some participants in the clinical group exhibited longer fixations and less efficient visual exploration, these patterns were not consistently observed across measures or individuals. This suggests that basic mechanisms of visual exploration may remain relatively preserved, with differences emerging primarily at the level of attentional efficiency and task-relevant prioritization rather than in oculomotor control itself. This interpretation is consistent with previous findings indicating that Alzheimer’s disease is associated with altered visual search strategies and reduced attentional efficiency, rather than a generalized breakdown of eye movement control [19,20,21].

Several factors may account for the relatively subtle group differences observed in the present study. First, the use of a free-viewing paradigm reduces task constraints and may therefore be less sensitive to deficits in goal-directed attention than more structured paradigms (e.g., visual search or anti-saccade tasks). Second, variability in eye-tracking data quality, particularly in older clinical populations, may have attenuated measurable group effects. Third, visuospatial deficits in dementia may be heterogeneous and context-dependent, becoming more pronounced in tasks that explicitly require integration of spatial information and goal-directed behavior.

Given their modest discriminative power in this paradigm, eye-tracking measures may be more informative when interpreted in conjunction with other modalities rather than as standalone indicators. Importantly, gaze-based measures do not isolate purely visuospatial processes. Alterations in eye movement patterns may reflect a combination of factors, including reduced attentional control, impaired novelty detection, slower processing speed, and executive dysfunction. The present findings therefore support an interpretation of eye-tracking differences as reflecting a broader visuospatial–attentional impairment, rather than a deficit in a single cognitive mechanism.

### 4.4. Multimodal Integration

The central contribution of the present study lies in demonstrating that dementia-related impairments are most clearly characterized when multiple cognitive domains are considered jointly. While individual measures revealed domain-specific deficits of varying magnitude, their integration across participants exposed a consistent and coherent pattern of multidomain impairment in the clinical group.

This finding is particularly relevant in light of the well-documented heterogeneity of dementia. Individuals with a similar clinical diagnosis often show uneven cognitive profiles, with relatively preserved performance in some domains and pronounced deficits in others. In such cases, single-domain assessments may underestimate impairment or fail to detect early-stage changes. The present results suggest that multimodal approaches can mitigate this limitation by capturing converging deviations across complementary cognitive systems.

From a methodological perspective, the multimodal approach also mitigates limitations associated with multiple comparisons. While individual measures may show variable sensitivity, the convergence of effects across domains provides a more stable and reliable indicator of group differences. This strengthens the interpretability of the findings in an exploratory context, where emphasis is placed on patterns rather than isolated statistical results.

Importantly, the observed multimodal pattern was not driven uniformly by all measures. Linguistic and behavioral variables showed the strongest and most consistent group differences, whereas eye-tracking measures revealed more heterogeneous and subtle effects. Rather than weakening the multimodal approach, this asymmetry provides important insight: different modalities appear to capture distinct aspects of cognitive decline, with varying sensitivity depending on task demands and underlying processes. The integration of these modalities therefore enables a more nuanced characterization of impairment than any single measure alone.

From a cognitive perspective, the results suggest that dementia-related deficits are most pronounced in tasks requiring structured processing, integration of information, and goal-directed behavior. In contrast, more basic perceptual and oculomotor processes appear relatively preserved. This pattern supports the view that higher-order cognitive control mechanisms are affected earlier and more consistently than lower-level sensory processes, consistent with models of distributed neurodegeneration affecting frontoparietal and temporoparietal networks.

While prior work suggests that multimodal approaches (especially the ones combining behavioral, linguistic, and physiological signals) have been proposed as potentially improving detection of cognitive decline [22,23], the present findings should be interpreted as a prerequisite step for evaluating whether such approaches could be informative in earlier stages. The current study demonstrates that multimodal profiling can capture coherent patterns of impairment in individuals with clinically established dementia. Whether these patterns extend to earlier or preclinical stages remains an open question that requires direct investigation in longitudinal or at-risk cohorts. Extending these approaches, the present study demonstrates that combining linguistic, behavioral, and eye-tracking measures can reveal robust cross-domain patterns of impairment using relatively low-burden, non-invasive measures. In this way, the present findings align with previous work emphasizing the value of multimodal approaches in dementia research.

Taken together, these findings suggest that multimodal profiling offers a promising avenue for improving the sensitivity and ecological validity of cognitive characterization in dementia. Rather than relying on single indicators, future diagnostic approaches may benefit from integrating complementary measures that jointly capture the distributed and heterogeneous nature of cognitive decline.

From a clinical perspective, it is important to clarify the intended role of the present multimodal protocol. In its current form, the approach is not designed to replace standard clinical assessment in patients with mild-to-moderate dementia, where established neuropsychological testing, functional evaluation, and clinical judgment already provide robust diagnostic information. Instead, the present study should be understood as a research-oriented framework aimed at characterizing how impairments manifest across interacting cognitive domains within individuals.

The multimodal index and associated measures are therefore not proposed as diagnostic tools in this severity range, but as a means of capturing distributed patterns of impairment that may not be fully apparent in standard domain-specific tests administered in isolation. In particular, the integration of linguistic, behavioral, and visual-attentional measures within related task contexts allows for the examination of how different cognitive systems interact during complex, goal-directed activities.

Accordingly, the current study represents a proof-of-concept step in a staged research program: first establishing the presence of coherent multimodal differences in clinically manifest dementia, before evaluating whether similar patterns can be detected in earlier or at-risk populations. Further work is required to determine whether such approaches provide incremental clinical value beyond existing assessment methods, and to assess their feasibility and utility in real-world clinical settings. It should also be noted that the multimodal protocol is relatively time- and resource-intensive, which may limit its immediate applicability in routine clinical settings and further supports its current role as a research tool rather than a standardized clinical instrument.

### 4.5. Statistical Considerations

An important consideration concerns the number of statistical comparisons conducted across multiple domains. Given the exploratory design and the broad range of outcome measures, individual statistical tests are subject to an increased risk of Type I error (false positives). For this reason, the present study does not interpret single *p*-values as definitive evidence but instead emphasizes the consistency and convergence of effects across measures.

Sensitivity analyses using false discovery rate (FDR) correction indicated that effects in linguistic and behavioral domains were largely stable, whereas eye-tracking effects were more strongly attenuated. This pattern further supports the interpretation that multimodal integration provides a more descriptive summary than individual measures. These considerations reinforce the interpretation of the findings as hypothesis-generating and highlight the importance of replication in larger, confirmatory studies.

A further consideration concerns the interpretation of effect sizes in small samples. Although non-parametric effect size estimates (r) are appropriate for skewed data and small sample sizes, it is well established that effect sizes derived from very small samples (such as the present N = 10 per group) are subject to substantial sampling variability and are often systematically inflated, a phenomenon sometimes referred to as the “winner’s curse” or Type M (magnitude) error. As a result, the effect sizes reported in this study should not be interpreted as precise estimates of the true population effects, but rather as descriptive indicators of the relative magnitude and direction of group differences within this sample. This limitation applies independently of statistical significance and underscores the need for replication in larger samples to obtain more stable and generalizable estimates.

### 4.6. Limitations and Future Directions

Several limitations should be considered when interpreting the present findings. First, the sample size is relatively small, which limits statistical power. As no a priori power calculation was conducted and the sample included only ten participants per group, the study should be interpreted as preliminary. While the multimodal design provides rich within-participant information, it does not overcome limitations related to imprecise effect-size estimates. In addition, the number of statistical tests increases the risk of false positives. Accordingly, results are interpreted with emphasis on consistency across measures rather than individual tests. Although this study adopts an exploratory framework and emphasizes cross-measure consistency, individual results should be interpreted with caution.

Second, the clinical group included participants with Alzheimer’s disease as well as unspecified dementia, or senile dementia (ADRD), introducing diagnostic heterogeneity that may contribute to variability in the observed patterns. Due to limited diagnostic detail, subgroup analyses were not feasible. The findings should therefore be interpreted as reflecting a mixed dementia sample rather than being specific to Alzheimer’s disease. The reliance on routine clinical diagnoses without standardized research-level confirmation or biomarker data reflects real-world practice but may reduce diagnostic precision. Future studies should include more homogeneous samples or directly compare dementia subtypes.

A related limitation concerns disease stage. Participants had mild to moderate dementia, and the findings therefore characterize established impairment rather than early or preclinical stages. Although multimodal approaches may be sensitive to early changes, this was not tested here. Longitudinal studies including at-risk populations are needed to determine whether similar patterns emerge prior to diagnosis.

An additional limitation concerns comorbid depression. Caregivers indicated that four clinical participants had a diagnosis of depression, but this information was not systematically linked to individual identifiers and could not be included in the analyses. Depressive symptoms may influence performance through decreased task engagement or motivation, psychomotor slowing (affecting time-based measures), and effects on language production, such as reduced verbal output and discourse coherence. While some observed differences could be partly influenced by mood, the overall pattern (particularly increased grammatical errors, pragmatic deviations, and task-specific failures) suggests that the findings cannot be explained solely by depression. Moreover, depression-related slowing would be expected to affect performance globally, whereas the present data show domain-specific patterns. Future studies should explicitly record and model such comorbidities.

The control group also presents limitations. Although participants were age-matched and screened for cognitive impairment, other relevant factors (e.g., education, cognitive reserve, lifestyle) were not systematically assessed. Moreover, cognitively healthy older adults who volunteer for research studies often represent a particularly active and cognitively engaged subset of the population. As a result, comparisons between clinical participants and highly functioning controls may either exaggerate group differences or obscure more subtle patterns of decline.

Gender imbalance represents another potential confound. The clinical group was predominantly female, whereas the control group included more males. Although not statistically significant, this imbalance may influence results, as sex differences have been reported in language, visuospatial abilities, and dementia presentation. While the direction of this imbalance might attenuate group differences in some domains (especially the linguistic domain), its overall impact cannot be determined. Future studies should aim for more balanced samples or explicitly model gender effects.

The gist measure should also be interpreted cautiously, as one control participant failed to identify the central theme. This likely reflects individual variability and highlights that single discourse measures are not sufficiently specific for clinical interpretation.

Finally, the fixed order of behavioral tasks may have introduced order effects such as fatigue or practice, which are especially relevant in clinical populations with reduced cognitive reserve. Although the largest group differences occurred in the final task, differences were already present earlier, suggesting that the pattern cannot be explained solely by order effects. Instead, the increasing magnitude of differences is consistent with higher cognitive demands in later tasks, although an additional contribution of fatigue cannot be excluded. Nevertheless, future studies should use counterbalanced designs to disentangle these influences more systematically.

Future research should aim to replicate these findings in larger and more homogeneous cohorts and employ longitudinal designs to assess whether multimodal profiles can predict cognitive decline. More fine-grained eye-tracking analyses (e.g., scanpath dynamics or entropy measures) may further clarify the role of visual attention and underlying cognitive mechanisms.

## 5. Conclusions

The present study demonstrates that cognitive impairment in Alzheimer’s disease and related dementias manifests across multiple domains, including language, behavior, and visual attention, rather than within a single isolated measure. A multimodal approach integrating linguistic, behavioral, and eye-tracking data revealed a consistent pattern of impairment in the clinical group, supporting the value of cross-domain assessment for capturing the multifaceted nature of cognitive decline.

While linguistic and behavioral measures showed robust group differences, eye-tracking measures reflected more subtle alterations in visual exploration, suggesting that different modalities capture distinct aspects of impairment. This complementarity highlights the advantage of combining multiple measures, as no single domain fully characterizes the observed deficits. These results suggest that multimodal assessment may offer greater sensitivity than single-domain approaches for capturing heterogeneous patterns of cognitive impairment. However, the present findings are limited to mild-to-moderate stages of dementia, and future longitudinal studies are required to determine whether such multimodal profiles can capture earlier or preclinical changes.

Overall, the findings indicate that cognitive differences in Alzheimer’s disease and related dementias are present across multiple domains rather than confined to isolated functions. While individual measures varied in sensitivity, the most consistent patterns were observed in linguistic and behavioral domains, with more variable contributions from eye-tracking measures.

These results should be interpreted within an exploratory framework and require confirmation in larger samples. Future work should examine whether similar cross-domain patterns can be detected in earlier stages of cognitive decline and whether multimodal profiling provides incremental value over established assessment approaches.

## Figures and Tables

**Table 1:** Demographic information about participants. Participants have been numbered according to ascending age. The clinical diagnoses in the clinical group included Alzheimer’s disease, Alzheimer’s dementia, and unspecified or senile dementia.

Clinical	Age	Gender	Diagnosis	Control	Age	Gender	Diagnosis
**D01**	73	female	Alzheimer’s	**H01**	75	male	none
**D02**	73	female	Alzheimer’s	**H02**	76	male	none
**D03**	78	male	dementia	**H03**	77	female	none
**D04**	82	female	Alzheimer’s	**H04**	77	male	none
**D05**	85	female	senile dementia	**H05**	80	male	none
**D06**	88	female	dementia	**H06**	82	female	none
**D07**	89	female	senile dementia	**H07**	84	male	none
**D08**	89	male	Alzheimer’s	**H08**	86	male	none
**D09**	89	male	Alzheimer’s	**H09**	91	female	none
**D10**	91	female	dementia	**H10**	93	female	none

**Table 2:** Linguistic measures for the narrative task: Total utterances, subordinate utterances, grammatically incorrect utterances, pragmatically unrelated utterances, and understanding of the central theme of the story (the gist) being narrated. The values for the three middle variables represent proportions relative to total utterances.

Clinical	U	Sub/U	Gram/U	Prag/U	Gist	Control	U	Sub/U	Gram/U	Prag/U	Gist
**D01**	147	0.13	0.13	0.01	0	**H01**	64	0.16	0.08	0.00	1
**D02**	111	0.09	0.15	0.00	0	**H02**	30	0.10	0.10	0.00	0
**D03**	95	0.01	0.33	0.06	0	**H03**	53	0.09	0.15	0.00	1
**D04**	78	0.01	0.22	0.05	0	**H04**	92	0.15	0.07	0.02	1
**D05**	155	0.08	0.14	0.23	0	**H05**	95	0.04	0.19	0.00	1
**D06**	56	0.02	0.21	0.41	0	**H06**	102	0.05	0.15	0.03	1
**D07**	57	0.05	0.14	0.07	1	**H07**	97	0.09	0.11	0.00	1
**D08**	53	0.02	0.11	0.00	1	**H08**	73	0.05	0.07	0.00	1
**D09**	104	0.01	0.13	0.00	1	**H09**	122	0.11	0.09	0.01	1
**D10**	97	0.10	0.30	0.32	0	**H10**	121	0.04	0.04	0.00	1

**Table 3:** Decision times and error rates of participants in both groups for the object manipulation task.

Clinical	Time (s)	Mistakes	Control	Time (s)	Mistakes
**D01**	185	0	**H01**	24	0
**D02**	36	0	**H02**	42	0
**D03**	246	0	**H03**	59	0
**D04**	171	0	**H04**	41	0
**D05**	226	0	**H05**	140	0
**D06**	355	2	**H06**	120	0
**D07**	81	0	**H07**	52	0
**D08**	52	0	**H08**	35	0
**D09**	18	0	**H09**	121	0
**D10**	457	0	**H10**	33	0

**Table 4:** Decision times and error rates of participants in both groups for the recognition behavioral task.

Clinical	Time (s)	Errors	Control	Time (s)	Errors
**D01**	111	0	**H01**	16	0
**D02**	7	0	**H02**	12	0
**D03**	29	0	**H03**	25	0
**D04**	23	0	**H04**	15	0
**D05**	168	1	**H05**	23	0
**D06**	70	1	**H06**	11	0
**D07**	21	0	**H07**	4	0
**D08**	6	0	**H08**	31	0
**D09**	12	0	**H09**	58	1
**D10**	133	1	**H10**	11	0

**Table 5:** Results of the object reconstruction act-out behavioral task.

Clinical	Time	Forfeits	Errors	Total	Control	Time	Forfeits	Errors	Total
**D01**	869	0	2	2	**H01**	92	0	0	0
**D02**	105	0	0	0	**H02**	90	0	0	0
**D03**	1265	2	1	3	**H03**	189	0	0	0
**D04**	351	0	1	1	**H04**	272	0	0	0
**D05**	1293	0	3	3	**H05**	432	0	1	1
**D06**	760	1	2	3	**H06**	473	0	1	1
**D07**	452	1	1	2	**H07**	197	0	0	0
**D08**	148	0	0	0	**H08**	114	0	0	0
**D09**	151	0	0	0	**H09**	1038	0	1	1
**D10**	992	1	2	3	**H10**	237	0	0	0
$\bar{x}$	638.60			1.70	$\bar{x}$	313.40			0.30
SD	458.75			1.34	SD	286.71			0.48

**Table 6:** Classification of mistakes in the reconstruction act-out behavioral task.

Target Confusion	Random Assembly	Minimal Mistake
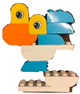	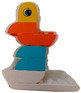	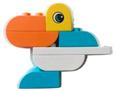
Blocks added to the target figure	Available blocks randomly stacked	Only one block incorrectly placed
5 cases	3 cases	7 cases
Only clinical group	Only clinical group	Both groups

**Table 7:** Quality of eye-tracking ratio (tracking ratio and calibration measures).

	Clinical Patients			Healthy Controls	
Clinical	Tracking Ratio	Calibration (x/y)	Control	Tracking Ratio	Calibration (x/y)
**D01**	90.86	0.78/1.07	**H01**	93.76	0.38/0.23
**D02**	95.14	0.31/0.54	**H02**	61.87	0.35/0.23
**D03**	90.65	0.19/0.44	**H03**	77.52	0.49/0.67
**D04**	71.79	0.46/0.69	**H04**	91.90	0.24/0.23
**D05**	98.11	3.74/5.91	**H05**	87.30	0.30/0.28
**D06**	51.14	0.58/0.75	**H06**	89.87	0.44/1.11
**D07**	87.85	0.73/0.32	**H07**	69.38	0.75/0.87
**D08**	76.92	0.20/0.80	**H08**	96.50	0.71/0.78
**D09**	89.72	0.75/1.99	**H09**	46.99	0.74/0.78
**D10**	93.81	1.63/1.21	**H10**	69.71	0.73/1.36
$\bar{x}$	84.60	0.94/1.37		78.48	0.51/0.65
SD	14.27	1.07/1.67		16.24	0.20/0.40

**Table 8:** Number of abnormally long fixations per participant for the landscapes.

Clinical	Canada	Chile	Total Long Fix.	Control	Canada	Chile	Total Long Fix.
**D01**	4	0	4	**H01**	2	3	5
**D02**	1	3	4	**H02**	7	2	9
**D03**	0	0	0	**H03**	0	1	1
**D04**	0	2	2	**H04**	3	0	3
**D05**	2	6	8	**H05**	1	1	2
**D06**	3	0	3	**H06**	5	2	7
**D07**	4	0	4	**H07**	2	0	2
**D08**	3	1	4	**H08**	1	0	1
**D09**	1	2	3	**H09**	2	0	2
**D10**	4	7	11	**H10**	1	0	1
$\bar{x}$			4.30				3.30
SD			3.09				2.79

**Table 9:** Entry times (ms) into the built objects AOIs for the scenes showing buildings in a natural setting.

Clinical	Castle	Cabin	$\bar{x}$ (ms)	Control	Castle	Cabin	$\bar{x}$ (ms)
**D01**	268.6	12.3	140.5	**H01**	3.3	4.4	3.9
**D02**	952.2	14.4	483.3	**H02**	320.1	15.8	168.0
**D03**	236.5	300.1	268.3	**H03**	322.6	554.9	438.8
**D04**	7.7	526.0	266.9	**H04**	5.1	13.6	9.4
**D05**	7437.6	1958.9	4698.3	**H05**	9.1	13.6	11.4
**D06**	7493.4	5.5	3749.5	**H06**	1.4	10.6	6.0
**D07**	266.8	4716.9	2491.9	**H07**	323.8	469.8	396.8
**D08**	232.6	12.2	122.4	**H08**	234.5	2.9	118.7
**D09**	13.3	2.1	7.7	**H09**	3.7	1411.3	707.5
**D10**	51.8	323.6	187.7	**H10**	13.0	842.0	427.5
$\bar{x}$			1241.6				228.8
SD			1743.9				247.8

**Table 10:** Participants who looked at the vanishing point AOI in the built stimuli.

Clinical	Dijon	Montpellier	Successes	Control	Dijon	Montpellier	Successes
**D01**	1	1	2	**H01**	1	1	2
**D02**	1	1	2	**H02**	0	1	1
**D03**	1	1	2	**H03**	1	1	2
**D04**	1	1	2	**H04**	1	1	2
**D05**	1	0	1	**H05**	1	1	2
**D06**	0	1	1	**H06**	0	1	1
**D07**	0	0	0	**H07**	1	1	2
**D08**	1	1	2	**H08**	1	1	2
**D09**	1	1	2	**H09**	0	1	1
**D10**	0	0	0	**H10**	1	1	2
$\bar{x}$			1.4				1.7

**Table 11:** Participants who looked at the white space AOI in the object stimuli.

Clinical	Cups	Hoodie	Total	Control	Cups	Hoodie	Total
**D01**	1	0	1	**H01**	1	0	1
**D02**	0	1	1	**H02**	0	0	0
**D03**	1	1	2	**H03**	1	1	2
**D04**	0	0	0	**H04**	1	0	1
**D05**	1	1	2	**H05**	1	0	1
**D06**	0	0	0	**H06**	0	1	1
**D07**	0	1	1	**H07**	1	0	1
**D08**	1	0	1	**H08**	1	0	1
**D09**	0	1	1	**H09**	0	0	0
**D10**	0	1	1	**H10**	1	0	1
$\bar{x}$			1.00				0.90

**Table 12:** Uncorrected and FDR-adjusted *p*-values (Benjamini–Hochberg) applied separately within each domain. Results surviving FDR correction (*p* < 0.05) are marked with *.

Domain	Measure	*p* (Uncorrected)	*p* (FDR)	Sig.
Linguistic	Total utterances	0.019	0.024	*
	Subordinate utterances	0.027	0.027	*
	Grammatical errors	0.010	0.017	*
	Pragmatic deviations	0.004	0.010	*
	Gist (Fisher’s exact)	0.019	0.024	*
Behavioral	Manipulation time	0.011	0.022	*
	Recognition time	0.021	0.025	*
	Reproduction time	0.016	0.024	*
	Reproduction errors	0.017	0.024	*
	Total failures	0.012	0.022	*
	Forfeits	0.087	0.087	
Eye-tracking	Long fixations	0.29	0.29	
	Foreground entry time	0.19	0.253	
	Vanishing point	0.18	0.253	
	White space	0.84	0.84	

**Table 13:** Number of dementia-markers across modalities measured for each participant in the sample.

Clinical	Dementia-Markers	Control	Dementia-Markers
**D01**	10	**H01**	1
**D02**	5	**H02**	3
**D03**	12	**H03**	4
**D04**	7	**H04**	2
**D05**	14	**H05**	6
**D06**	14	**H06**	9
**D07**	10	**H07**	1
**D08**	2	**H08**	2
**D09**	3	**H09**	10
**D10**	12	**H10**	3

## Data Availability

Some stimuli, materials, raw data and participants responses can be accessed at osf.io/ev2u6. Due to copyright restrictions, the adapted picture-book stimuli cannot be publicly shared. Researchers may consult the original publication, and task structure/coding materials are available on OSF.

## Abbreviations

The following abbreviations are used in this manuscript:

AD	Alzheimer’s disease
ADRD	Alzheimer’s disease and related dementias
SCI	Subjective Cognitive Impairment
MMSE	Mini-Mental State Examination
MoCA	Montreal Cognitive Assessment
AOI	Area of Interest
VR	Virtual Reality
MRI	Magnetic Resonance Imaging
Md	Median
SD	Standard Deviation
